# Surpassing the nonlinear conversion efficiency of soliton microcombs

**DOI:** 10.1038/s41566-023-01280-3

**Published:** 2023-08-31

**Authors:** Óskar B. Helgason, Marcello Girardi, Zhichao Ye, Fuchuan Lei, Jochen Schröder, Victor Torres-Company

**Affiliations:** Department of Microtechnology and Nanoscience, Chalmers University of Technology, Gothenburg, Sweden

**Keywords:** Frequency combs, Solitons, Nonlinear optics

## Abstract

Laser frequency combs are enabling some of the most exciting scientific endeavours in the twenty-first century, ranging from the development of optical clocks to the calibration of the astronomical spectrographs used for discovering Earth-like exoplanets. Dissipative Kerr solitons generated in microresonators currently offer the prospect of attaining frequency combs in miniaturized systems by capitalizing on advances in photonic integration. Most of the applications based on soliton microcombs rely on tuning a continuous-wave laser into a longitudinal mode of a microresonator engineered to display anomalous dispersion. In this configuration, however, nonlinear physics precludes one from attaining dissipative Kerr solitons with high power conversion efficiency, with typical comb powers amounting to ~1% of the available laser power. Here we demonstrate that this fundamental limitation can be overcome by inducing a controllable frequency shift to a selected cavity resonance. Experimentally, we realize this shift using two linearly coupled anomalous-dispersion microresonators, resulting in a coherent dissipative Kerr soliton with a conversion efficiency exceeding 50% and excellent line spacing stability. We describe the soliton dynamics in this configuration and find vastly modified characteristics. By optimizing the microcomb power available on-chip, these results facilitate the practical implementation of a scalable integrated photonic architecture for energy-efficient applications.

## Abstract (web summary)

Researchers demonstrated coherent dissipative Kerr solitons with a conversion efficiency exceeding 50% and good line spacing stability. The results may facilitate practical implementation of a scalable integrated photonic architecture for energy-efficient applications.

## Main

**Fig. 1:**
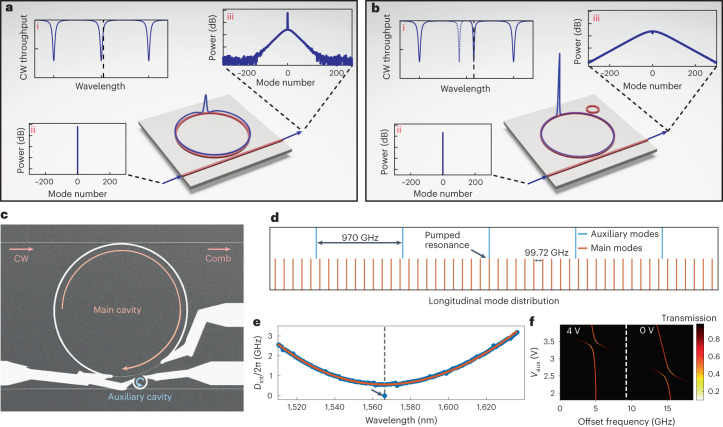
Concept of inducing a phase shift to the pump mode for power-efficient comb generation. **a**, Soliton generation in a single microring resonator fabricated on-chip, whose separation between resonances is defined by the cavity length and the anomalous-dispersion of the waveguides (i). The microring is pumped with a CW laser (ii), with the laser frequency effectively red-detuned from cavity resonance, as illustrated by the dashed line in (i). Such configuration can exhibit the generation of a temporal soliton, whose power distribution is displayed on top of the microring. Due to the high red-detuning, much of the CW laser bypasses the cavity, resulting in an output spectral envelope (iii) with a strong CW component and low comb power^17^. The *y*-axes in (ii) and (iii) have 20 dB div^–1^ scaling. **b**, The same microring now has a mechanism that causes a constant phase shift each roundtrip to the pump mode only, for example, by inducing a mode-crossing using linear coupling to a small auxiliary cavity. This causes the pump resonance to appear shifted while other resonances remain in the same place (i). The pump can now be shifted further towards the red side while still coupling ample amount of power into the cavity. This results in higher soliton power and comb power (iii) while operating with a CW laser. **c**, A representative image of a fabricated photonic molecule taken with a scanning electron microscope. The white tracks are metallic heaters. **d**,**e**, Illustration of the modal distribution of the two cavities, where the mismatch in FSR allows a strong mode-shift to be applied to one longitudinal mode at a time (**d**). This is reflected in the dispersion measurement (**e**), which shows a relatively smooth anomalous profile of the main cavity with a strong shift at the pump mode (marked by the arrow) induced by coupling to the auxiliary cavity. **f**, Shows that the mode-crossing induced by the auxiliary ring can be controlled by tuning the auxiliary heater voltage (*V*_aux_) and through the main heater voltage (set to 0 V and 4 V).

Driven by a continuous-wave (CW) laser, dissipative Kerr soliton (DKS) microcombs are maintained through balancing optical losses with parametric gain, and dispersion with Kerr nonlinearity in a high-Q microresonator^1^. Dissipative Kerr solitons result in a train of optical pulses coupled out of the microcavity, which corresponds to a coherent optical frequency comb in the spectral domain. Such soliton microcombs present new opportunities of relevance in optics^2–4^, such as telecommunications^5^, lidar^6^, optical frequency synthesis^7^, microwave photonics^8,9^, calibration of astronomical spectrographs^10^ and quantum optics^11^.The bulk of DKS studies has been conducted using single cavities. In an optical resonator displaying anomalous dispersion, the DKS features a hyperbolic-secant profile^12,13^, whose coherence and robustness has been illustrated by multiple demonstrations. In the presence of CW bistability, these waveforms can be formed through modulational instability by tuning the CW laser from the blue side of the resonance towards the red^12,14^. The DKS can only be maintained with the laser effectively red-detuned (Fig. 1a), where the number of comb lines and generated comb power tends to increase with higher detuning^15,16^. Unfortunately, higher laser detuning reduces the amount of power coupled into the cavity. This imposes a fundamental limitation in the conversion efficiency, that is, the ratio of power converted from the input CW pump to other frequency components at the output^17^. The DKS conversion efficiency scales inversely with the number of lines generated^16^, leading to a fundamental trade-off between the spectral density and power per line. The low conversion efficiency places stronger requirements in the performance of on-chip lasers, interposers and frequency doublers for realizing self-referencing^18^—a key ingredient in modern frequency synthesis and metrology. Improving the conversion efficiency is instrumental to leveraging advances in photonic integration^19–21^ and realizing fully integrated microcomb-based systems on-chip.

The limited conversion efficiency problem is exclusive to single DKSs in anomalous dispersion microresonators. Other coherent microcomb states—such as soliton crystals^22–24^, Turing rolls^25^ or dark-pulse Kerr combs^21,26–28^—display much higher conversion efficiency, but they come with other caveats, such as a limitation in the number of lines or narrower bandwidths compared with single DKSs in anomalous-dispersion microresonators. Pulsed pumping improves the conversion efficiency, but this is more cumbersome from an integration perspective, as it requires a comb to generate a DKS microcomb^29^. Another proposal for boosting the conversion efficiency is to use an external cavity with optical gain^30^. However, the high efficiency of this solution has thus far been severely limited with regards to the number of comb lines. Other chip-scale comb generators such as electro-optic combs can reach high optical conversion efficiencies, but at the cost of requiring excess microwave driving power^31^.

New comb states and soliton dynamics have recently been discovered in coupled-cavity systems^32,33^. These photonic devices are often termed photonic molecules because their eigenfrequency distribution is akin to the energy levels in molecular and solid-state systems^34,35^. Microcombs generated in photonic diatomic molecules offer a path to high power conversion efficiency^36^, but the demonstration of efficient DKS microcombs in the anomalous dispersion regime has hitherto remained elusive. Ref. ^37^ demonstrates an arrangement with two linearly coupled cavities: one displaying normal dispersion, where the pump is fed, and another with anomalous dispersion, where the DKS is generated. The intermediate normal-dispersion cavity is described as a storage unit that recycles the pump, enabling high-efficiency comb generation. The pump recycling concept was experimentally demonstrated in fibre cavities with pulse-initiated DKSs, leading to an increase in soliton energy; however, to the best of our knowledge, high-efficiency single DKSs have not been experimentally achieved using this arrangement. Here we show a different configuration in which pump recycling is not strictly required for the enhancement in power conversion efficiency, but the principal mechanism is instead the shift induced to the pump resonance of the anomalous soliton cavity (Fig. 1b). Our numerical investigations show that by artificially shifting just the pump resonance of a single anomalous-dispersion cavity, it is possible to operate the soliton with the pump close to centre of resonance while other comb lines experience high red-detuning. Physically, this allows the pump to be coupled more efficiently into the cavity while fulfilling the red-detuned criteria of DKSs, resulting in DKS microcombs with a conversion efficiency approaching unity (see Fig. 1biii). Furthermore, the resulting comb state has more lines generated, which indicates that the inverse scaling of the conversion efficiency with the number of lines generated is overcome using this approach. As shifts on specific resonances can be attained in other photonic devices such as photonic crystal resonators^38^, we anticipate that high-efficiency solitons could be in principle attained in systems different to the photonic molecules discussed in this work. Our results provide the first demonstration of high-efficiency single DKS microcombs pumped by a CW laser, offering a clear pathway towards realizing practical systems that benefit from their exceptional bandwidth, spectral density and frequency stability.

## Results

### Controllable frequency shift in photonic molecules

We consider a photonic molecule arrangement of two linearly coupled cavities with anomalous dispersion and largely dissimilar volumes (Fig. 1c–f). The pump is coupled directly into the main cavity (larger ring) while the pump resonance is shifted with the aid of an avoided mode-crossing introduced via coupling to an auxiliary cavity (smaller ring). We fabricated such devices in silicon nitride using a subtractive processing method with heaters on top of the cavities^39^. The heaters allow us to control the location of the avoided mode-crossing and the strength of the coupling^35,40^, thus tuning the frequency shift of the pump resonance in a controllable manner. Both cavities have identical cross-sections, but the auxiliary cavity is shorter, resulting in a larger free spectral range (FSR) that minimizes the interaction between cavities at longitudinal modes other than the pump mode (Fig. 1d). To further minimize mode crossings at adjacent resonance locations, the FSRs of the auxiliary and main rings are not commensurate to each other. The result is a main cavity with anomalous dispersion, as indicated by the integrated dispersion (*D*_int_) in Fig. 1e with the auxiliary cavity shifting a single resonance location. The dispersion profile is similar to a previous study of a photonic crystal resonator^38^, except that our photonic molecule system has the flexibility of introducing a tunable shift (see Fig. 1f). By pumping the shifted resonance with a CW laser, we achieve a power conversion efficiency exceeding 50%, which is an order-of-magnitude improvement compared with previous experimental demonstrations in CW pumped anomalous dispersion microresonators^41^. We further unravel the pathway to the generation of power-efficient microcombs from a CW laser using a bidimensional existence map^15,42^. The analysis results in unexpected dynamics, such as the existence of DKSs on both the blue and red sides of the resonance, the possibility of backwards DKS initiation and DKS operation below modulational instability threshold, properties idiosyncratic to these pump-shifted cavities.

### Mapping the existence of DKSs with a shifted pump resonance

**Fig. 2:**
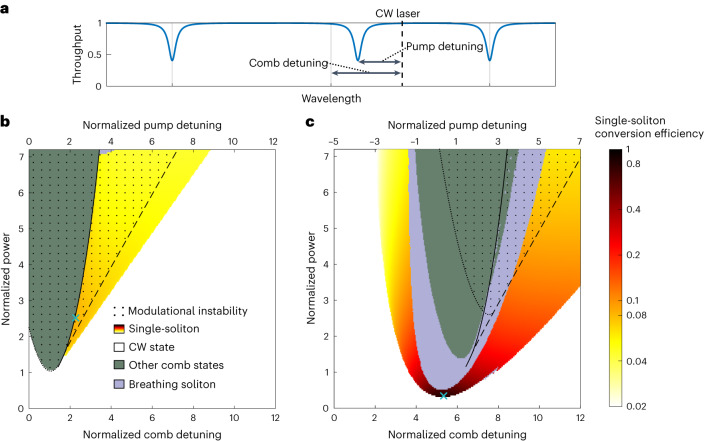
Existence maps showing the conversion efficiency of a single DKS state. **a**, A resonance profile with a shifted centre resonance, showing the definition of comb detuning and pump detuning. The two detuning parameters become identical when no shift is applied. **b**, Map for an anomalous dispersion cavity without shift applied to the pump resonance. The areas confined by the dashed lines indicate the presence of CW bistability. The dotted areas indicate where combs could be initiated through modulational instability. The peak conversion efficiency is 8% at a point indicated by the light blue cross on the map. **c**, An existence map where the pump resonance has been shifted by five normalized units. The peak conversion efficiency is 80% at the point indicated by the light blue cross in the map.

To investigate the impact of shifting the pump resonance in an anomalous dispersion cavity, we conduct a numerical investigation of the existence map of DKSs in terms of CW pump power and laser detuning. The main objective is to map the conversion efficiency of single DKSs and the presence of modulational instability (MI). Other comb states that do not correspond to a single DKS (Turing rolls, chaos, multi-DKSs) were also observed, but their detailed characterization is left for a later study. The dynamics of the system is simulated with an Ikeda map^43^, with the pump resonance shift induced by an extra phase shift applied to the pump frequency each roundtrip^26^. Effectively, this leads to two different detuning factors: the detuning between the CW pump frequency and the shifted resonance (defined as pump detuning); and the detuning experienced by the other DKS frequencies (defined as comb detuning), see Fig. 2a. Similar to previous investigations of the unaltered anomalous-dispersion cavity^15,42^, we use the mean-field approximation to plot the existence map in terms of normalized power (*X*), normalized comb detuning (*Δ*_c_) and normalized pump detuning (*Δ*_p_) (see Methods for definitions).

We begin the analysis by considering a microresonator with zero phase shift applied to the pump (Fig. 2b). Bistability is found in the area confined by the solid and dashed lines, where the solid line marks the up-switching point^44^. The distribution of comb states across the existence map is in agreement with past works^42,45^. As expected for standard anomalous-dispersion cavities, the power conversion efficiency is rather low, limited to the range of 4–8%. The absolute maximum conversion efficiency is found to be 8% at a location of roughly 2.5 normalized power units with the corresponding DKS waveforms plotted in Fig. 1a. Modulational instability is found on the blue side of the CW bistability above a threshold near *X* *=* 1, as previously predicted^46^.

In Fig. 2c we consider the same cavity, but with the pump resonance shifted by five normalized detuning units. Now, single DKSs exist in a continuous area that stretches between the red and blue sides of the pump resonance—a noteworthy result considering that DKSs can only exist on the red side of pump resonance in standard anomalous-dispersion cavities. This blue detuning operation is in agreement with the simulated findings in photonic crystal resonators^38^. The reason for DKSs existing with a blue-detuned pump is the fact that the DKS comb lines experience red-detuning, that is, the comb detuning always has a positive value. Our study of the closed-form DKS solution shows that the comb detuning determines the shape of the DKS (see Supplementary Section 1). This suggests that the detuning of the DKS is in fact determined by the comb detuning rather than the pump detuning.

The conversion efficiency can now easily reach double digit percentage. Such an enhancement is due to a twofold effect attained by the local frequency shift induced on the pump. On one hand, for a fixed pump power, the frequency shift allows the soliton to exist at much larger comb detuning values compared with the unperturbed cavity. Operating at large comb detuning values enhances the soliton energy and bandwidth^15,16^. The second effect is seen by noting that solitons can exist in a region in which the effective pump detuning is zero, so that the pump power is efficiently coupled into resonance and allowing the comb to be operated at a lower input power. The combined effect results in a dramatic improvement in power conversion efficiency (80% for parameters *Δ*_c_ = 5.32 and *X* = 0.32), which could approach unity if the resonance is shifted further. In Supplementary Section 1, we extend the analysis of the high-efficiency DKSs using the closed-form solutions of the soliton electric field amplitude, showing that our approach overcomes the fundamental scaling law of nonlinear conversion efficiency with the number of lines generated.

The single DKS shares a boundary with breather states^47^, which were not present at such low power levels in Fig. 2b. Other states appear at the inner boundary of the breather states, which we observe to be mostly chaotic. These can be initiated from modulational instability near the bistability region of the pump resonance. The modulational instability threshold has been shifted from *X* = 1 of the unperturbed cavity to above *X* = 2.5, which is a consequence of the high comb detuning impeding the phase-matching condition required for modulational instability (see Supplementary Section 2). This is in sharp contrast to normal dispersion microcombs, where the phase matching is enhanced by such resonance shifts^35^. This absence of modulational instability at lower power levels might be the reason the DKS is allowed to exist at the centre of resonance at low power, which is an effect not found in unaltered anomalous-dispersion cavities.

The excitation pathway leading to the formation of DKSs from a CW laser has been radically changed due to the presence of the controllable frequency shift on the pump mode. The fact that the maximum efficiency DKS is located far below the modulational instability threshold of *X* = 1 means that the input power will have to be lowered after initiation. Also, to minimize the initiation power, the initiation should be performed with an unshifted pump resonance, with the shift only applied once a comb has been generated. These aspects are utilized as we study the initiation of high-efficiency solitons in photonic molecules in the next sections.

### From continuous-wave to power-efficient solitons

To experimentally demonstrate a high-efficiency DKS, we use a device with a layout similar to Fig. 1c,d. The FSR is 99.79 GHz and the group velocity dispersion (GVD) coefficient is *β*_2_ = −81 ps^2^ km^–1^. The FSR of the auxiliary cavity was 967 GHz. The heaters of the cavities were set such that a resonance split appears near 1,565.2 nm (similar to Fig. 1f). The red-shifted hybridized resonance was pumped with a CW laser to generate the DKS state.

**Fig. 3:**
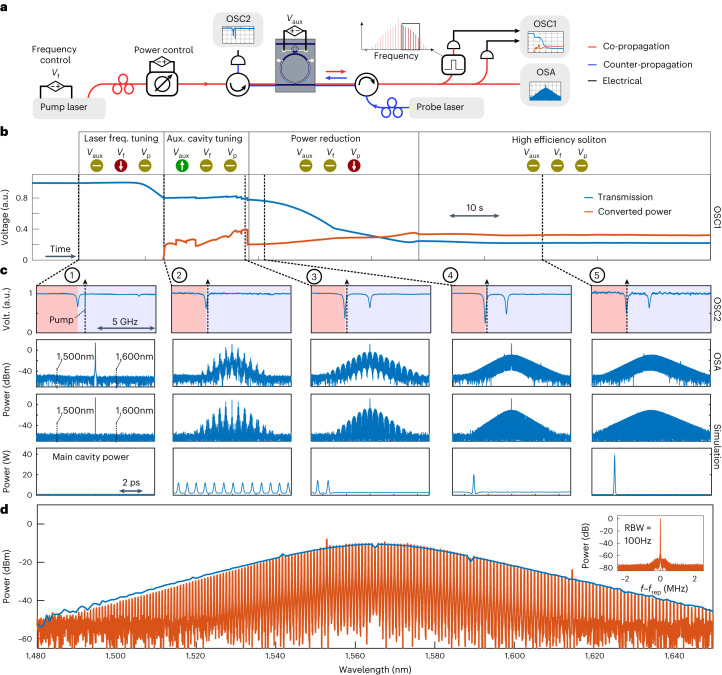
Pathway to power-efficient DKS microcombs. **a**, Set-up used to control and monitor the high-efficiency soliton initiation. The pump laser generates a comb in the main cavity, whereas the probe laser is scanned in frequency to spectrally characterize the pumped resonance. **b**, Evolution of the converted power and transmitted power over time measured in oscilloscope 1 (OSC1). The three tuning steps (laser frequency, auxiliary heater and input power) used to reach a high-efficiency soliton are indicated, with arrows indicating the tuning direction of each control signal. The converted power shows where a comb is generated, with modulational instability states and multi-solitons found in the first 20 s of the converted trace, whereas a single soliton state was found after transition near the end of the heater tuning step. **c**, Selected comb states in the initiation process, marked by dashed lines in **b**. The top row shows the resonance scan from the counterpropagating probe measured by the oscilloscope OSC2, with the *x*-axis showing frequency change and the shading indicating the blue and red sides of the pumped resonance. The beat note with the pump laser shows that the pump laser is consistently located at the blue side of the resonance throughout the initiation process. The second row shows the corresponding comb state measured in the optical spectrum analyser (OSA). The third and fourth rows show the output spectrum and the main cavity temporal power found through numerical simulations, qualitatively replicating the initiation process. **d**, A highlighted version of the final soliton, showing an experimental (red) and simulated on-chip output spectrum. The inset shows the measured beat note between comb lines, acquired by electro-optic downconversion and measured with a resolution bandwidth (RBW) of 100 Hz. The final measured (simulated) spectrum has 54% (48%) conversion efficiency.

The process of initiating a single DKS in our device is fundamentally different from the standard approach using a single anomalous cavity, as we find that the pump laser frequency can be maintained consistently on the blue side of the pumped resonance throughout the DKS initiation process. This contrasts with the unperturbed anomalous cavity, where the DKS state only exists on the red side of resonance. To show this, we use a set-up (see Fig. 3a) that allows us to generate a microcomb using a pump laser, while a counterpropagating probe laser is scanned in frequency to spectrally characterize the location of the pump laser with regards to the resonances^48^. The soliton initiation involved a series of steps in which we slowly tuned the pump laser frequency, the auxiliary cavity heater and laser power over a long-time span using input control voltage signals. These steps are displayed in Fig. 3b, showing evolution of the converted power and transmitted power. Figure 3c shows the counterpropagating resonance scan and the spectral power of the microcomb output for individual comb states (labelled 1–5) found at different points in this scan. We start with the pump laser on the blue side of the pumped resonance with 13.5 dBm of power, with no comb being generated, as shown by state 1. The auxiliary mode was set further on the blue side, with limited interaction with the pumped resonance. The first tuning step is to reduce the pump frequency (reduce *V*_f_), tuning the laser into resonance until a Turing roll is generated, as indicated by comb state 2. Now that a comb has been initiated, the auxiliary heater voltage (*V*_aux_) is increased to shift the auxiliary resonance to a lower frequency, closer to the pumped resonance, which results in a mode-split that shifts the pumped resonance further to the red side. During this step, the Turing roll evolves into a multi-DKS state (see comb state 3), with the number of solitons in the cavity reducing step-by-step until a single soliton is left (see comb state 4). Finally, we reduce the pump power (*V*_p_) to 8.7 dBm (depending on the device, additional adjustments might also be required to the laser frequency and auxiliary cavity). In the resulting final state 5, we achieve a DKS with more than 54% conversion efficiency, with the blue-detuned pump close to resonance centre. This initiation process was found to be very stable and could be achieved by tuning the instruments by hand. This is possible as the pump remained located at the thermally stable blue-side throughout the initiation process. Thus, the pump acts as a stabilizer for the cavity, which would typically require an additional laser when pumping a single anomalous cavity^49,50^. The initiation was found to be reliable at slow tuning speeds as discussed in Supplementary Section 4.

A numerical simulation of two coupled cavities was conducted using parameters similar to the characteristics of our device, qualitatively replicating the initiation process (see Fig. 3c and Supplementary Section 5). The simulation reaches a high-efficiency single soliton in the final state, with 48% efficiency.

The final DKS comb state is highlighted in Fig. 3d and it features a smooth sech spectrum, exhibiting 54% conversion efficiency in the experimental measurement. The inset shows a measurement of the comb’s repetition rate measured through electro-optic downconversion^51^, indicating a coherent comb state.

Much of the DKS initiation process in Fig. 3 can be understood from the analysis in Fig. 2. The first comb states were initiated with a small shift to the pump resonance, as it allows modulational instability to occur at a lower power level, with a stronger shift applied after initiation. The comb initiation had to be performed at a higher power, with the power lowered after initiation as the most efficient comb exists below the modulational instability threshold. The blue-detuned existence of DKS states is also predicted by Fig. 2. In Supplementary Section 8, we experimentally identify more features from the existence map, including breather solitons, chaotic states, and a DKS transitioning between blue and red-detuning.

The DKS initiation is not limited to the scheme in Fig. 3. Initiation at a lower power (12.3 dBm) was possible, although it was more sensitive and required feedback locking between comb power and laser frequency^52^. A DKS state with a 35% conversion efficiency could be reached without reducing the power after initiation (see Supplementary Section 8). A single DKS could be initiated using backwards tuning of the laser frequency, a consequence of the DKS state existing on the blue side of modulational instability (see Supplementary Section 9).

A remarkable feature of the final comb state in Fig. 3d is the fact that more than 99% of the CW pump component has been depleted, such that it is below the spectral envelope of the DKS state. This is in high contrast to single-cavity DKS states that typically include a strong CW pump background. This occurs because the intrinsic losses of the pump frequency, that is, conversion to other comb lines, in combination with intrinsic losses in the main and auxiliary cavities, are at the same level as the coupling rate between the bus and cavity, which effectively induces a critically coupled state from the view point of the CW frequency^37^. Importantly, the large depletion of the CW component does not imply absence of continuous background in the intracavity field. In a numerical study (see Supplementary Section 7), we verify that such pump depletion would constitute an almost unity conversion efficiency in absence of intrinsic losses. In the same study, we found that high-efficiency solitons can exist in the presence of strong third-order dispersion, with a large tolerance to the coupling strength between cavities. These features indicate the robustness of high-efficiency DKS microcombs to the design characteristics.

The microcomb displayed high level of flatness, with 35 consecutive lines above −13 dBm and 73 consecutive lines above −20 dBm. Such level of flatness and efficiency is appealing in multiple applications, such as optical communications^5^. However, the requirements in terms of power-per-line and number of lines may differ between different application systems. In Supplementary Section 3, we both numerically and experimentally show how the DKS can be scaled in power and in number of lines generated, mainly by tuning the coupling factors and GVD.

### Highly coherent 50 GHz DKS

**Fig. 4:**
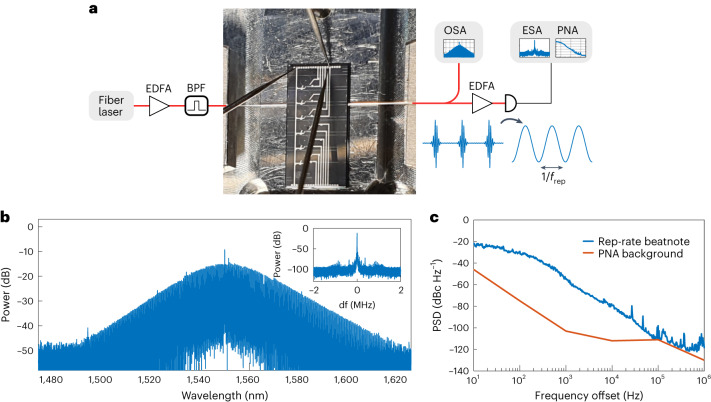
Highly efficient soliton microcombs and microwave frequency synthesis. **a**, A simplified diagram of the measurement set-up, including a picture of the optically coupled chip with power supplied to heater pads through positional probes. A high-speed photodiode transforms the output pulses into a microwave tone at a frequency equal to the repetition rate. **b**, The measured spectrum of a microcomb operated with 35% conversion efficiency. The inset shows the repetition-rate beat note at 49.85 GHz measured in an ESA. **c**, The power spectral density (PSD) of the repetition-rate beat note measured using a phase noise analyser (PNA).

In this section we demonstrate that highly efficient DKS microcombs can be operated with different cavity volumes. For this, we double the length of both the main and auxiliary cavities, resulting in an FSR of 49.85 GHz and 486.5 GHz, respectively. The DKS microcomb operates at a repetition rate commensurate with electronics bandwidth, which is relevant for applications in telecommunications^5,27^, radiofrequency photonics^8^ and the calibration of astronomical spectrographs^10^. Enhancing the cavity volume is challenging with planar integrated technologies, and the decrease associated with intracavity intensity must be encompassed with an increase in quality factor and pump power^53^. The large conversion efficiency allows us to relax these requirements while maintaining outstanding purity in the repetition rate frequency. A simplified schematic of the set-up is displayed in Fig. 4a, which also shows how the device was optically coupled using lensed fibres. The heaters were tuned such that the auxiliary cavity induced a resonance split near 1,550.1 nm. This split resonance was pumped with a low-phase-noise fibre-laser at a fixed wavelength of 1,550.1 nm. The output spectrum of the comb was characterized in an OSA (see Fig. 4b), resulting in a DKS microcomb with 35% conversion efficiency. The repetition rate of the comb was measured directly as a beat note using a high bandwidth photodiode connected to an electrical spectrum analyser (ESA). The result was a narrow tone located near 49.85 GHz with low power spectral density (see Fig. 4c), indicating a coherent comb state with a phase noise comparable with state-of-the-art DKS microcombs in single microresonators^9^.

## Discussion

We have demonstrated high-efficiency soliton microcombs generated in anomalous dispersion microresonators, enabled by shifting the CW pumped resonance of the microcavity. This shift was realized in practice using an avoided mode-crossing, induced by an auxiliary resonator coupled to the main cavity. We discovered that such modified cavities enabled a reliable DKS initiation process, with the CW laser blue-detuned from the pumped resonance, dynamics not found in single anomalous-dispersion cavities. We experimentally demonstrated three devices operating with different input powers, each exhibiting a smooth DKS spectrum with up to 54% conversion efficiency. To our knowledge, this is the highest conversion efficiency achieved for a single soliton state generated in a microresonator from a CW pump. This high conversion efficiency and uniform spectrum make these devices highly relevant for applications in optical communications^5^ and dual-comb spectroscopy^54^. With further engineering of the coupling region and dispersion, we believe these results pave the way for the realization of octave-spanning microcombs and self-referencing using only integrated components.

Finally, a shifted resonance can be achieved in multiple different systems, and is not limited to the coupled-cavity design presented here. As such, this work provides important insights for realizing high-efficiency solitons using other schemes, such as photonic crystal resonators^38^, or linearly coupled transvers modes^55^ and, potentially, when using a feedthrough pump cavity^37^.

## Methods

### Device characterization

The devices were fabricated on a chip using a silicon nitride waveguide core, with a silica cladding using a subtractive processing method^39^. Heaters were placed on both auxiliary and main cavities, using positional probes to connect voltage to the heaters. The devices were accessed by coupling light into the bus waveguide at the chip facets using a lensed fibre. Most of the devices were spectrally characterized using a self-referenced mode-locked laser frequency comb as a reference, in a manner similar to our previous work^36^. The conversion efficiency is characterized using an OSA to measure the output spectrum from the bus waveguide. First, we measure the output with a microcomb being operated, adding up the power of all comb lines excluding the pump frequency (*P*_comb,total_ – *P*_comb,CW_). We then turn off the comb by tuning the laser frequency completely out of resonance, and do a second measurement in the OSA, recording the power of the CW component (P_CW-ref_). The conversion efficiency is then calculated as (*P*_comb,total_ – *P*_comb,CW_)/*P*_CW-ref_.

The main manuscript involved two separate microcomb devices. Device 1, used in the experimental demonstration of Fig. 3, had a facet coupling loss of ~2 dB per facet. It had a main cavity of radius 227.26 µm, whereas the radius of the auxiliary cavity was 23.36 µm. All waveguides had the same dimensions of 1,800 nm width and 740 nm height. The gap between rings was 450 nm, the gap between main cavity and bus was 300 nm, and the gap between auxiliary cavity and bus was 670 nm. The main cavity was measured (referenced to 1,565 nm) with a GVD of *β*_2_ = −81 ps^2^ km^–1^, FSR of 99.79 GHz, intrinsic Q-factor with an average near 14 million, and a coupling rate to bus waveguide corresponding to extrinsic Q of 1–2 million near 1,565 nm. The maximal resonance shift induced by the auxiliary cavity was measured as 735 MHz. The auxiliary cavity had intrinsic Q of 0.6 million and coupling rate to a bus waveguide corresponding to 50 million of extrinsic Q.

Device 2, used in the experimental demonstration of Fig. 4, had a facet coupling loss of ~2 dB per facet. It had a main cavity of radius 455.02 µm and the radius of the auxiliary cavity was 46.62 µm. All waveguides had the same dimensions of 1,800 nm width and 740 nm height. The gap between rings was 450 nm, the gap between main cavity and bus was 350 nm, and the gap between auxiliary cavity and bus was 700 nm. The main cavity was measured (referenced to 1,550 nm) with GVD of *β*^2^ = −82 ps^2^ km, FSR of 49.86 GHz, intrinsic Q-factor with an average near 10 million, and a coupling rate to bus waveguide corresponding to extrinsic Q of 2–3 million near 1,550 nm. The maximal resonance shift induced by the auxiliary cavity was measured as 440 MHz. The auxiliary cavity resonance at 1,550, which interacted with the main cavity, had near four million intrinsic Q and coupling rate to a bus waveguide corresponding to 60 million of extrinsic Q. The auxiliary FSR was 486.5 GHz.

Using a mode-solver for the cross-section of the microresonator waveguides, the nonlinear parameter was estimated to be *γ* = 0.9 (W m)^−1^ for both devices.

The microcomb characterization of spectral distribution, conversion efficiency and repetition rate was conducted using the methods described in our previous work^36^, unless otherwise specified.

The combs in Figs. 3–4 were pumped with an external cavity diode laser, which was amplified with an erbium-doped fibre amplifier (EDFA). The trace in Fig. 3b was recorded using pre-programmed control voltages applied by a function generator (see Supplementary Section 4 for details). Measurements of individual comb states in Fig. 3c were recorded after tuning the comb by hand to states corresponding to the points labelled in Fig. 3b. The counterpropagating laser used in Fig. 3 was another external cavity diode laser operated at −5 dBm. The trace in Fig. 3c measured in oscilloscope 2 was low-pass filtered with a 0.5 MHz bandwidth such that a beat note would only appear near the pump laser frequency. The trace was scaled to remove a constant background coming from the reflected pump.

The measurement in Fig. 4 involved a low-phase-noise fibre-laser (NKT Koheras Basik), which operates at a wavelength of 1,550.1 nm. The laser was amplified with an EDFA, with the amplified spontaneous emission noise removed in a notch filter just before being coupled into the chip. A comb was initiated using a method similar to that of Fig. 3, only the detuning between laser and resonances was managed by changing the cavity heaters with the help of feedback locking using the comb power. An EDFA was used as a pre-booster for the beat note detection. The beat note was amplified in a high-speed electrical amplifier before detection in the ESA. The ESA was a PXA signal analyser N9030A, which has phase noise analysing functionality to measure the single-sideband PSD.

### Numerical models

The numerical simulation used in Figs. 1 and 2 is based on an Ikeda map^43^ modified to include a phase shift to the pump frequency^26,38^. Each roundtrip simulated for the optical field of the microring includes a coupling step, nonlinear propagation around the full length of the microring (L) and a phase shift applied only to the pump frequency. The coupling regime describes the coupling between a straight bus waveguide and a ring waveguide. It is approximated as point coupling given by the following equation:

$$\left[\begin{array}{c}{A}_{\text{out}}\\ A\end{array}\right]=\left[\begin{array}{cc}\sqrt{1-\theta } & {\rm{i}}\sqrt{\theta }\\ {\rm{i}}\sqrt{\theta } & \sqrt{1-\theta }\end{array}\right]\left[\begin{array}{c}{A}_{{\rm{in}}}\\ {A}^{{\prime} }\end{array}\right],$$

Where *A* is the temporal field of the ring cavity, ${A}_{\text{in}}$ is the input pump field in the bus waveguide, ${A}_{\text{out}}$ is the output field in the bus waveguide, and *θ* is the portion of power coupled between the bus and ring. The nonlinear propagation is described by the nonlinear Schrödinger equation

$$\left(\frac{\partial }{\partial z}+\frac{{\alpha }_{i}}{2}+i\phi +{\rm{i}}\frac{{\beta }_{2}}{2}\frac{{\partial }^{2}}{\partial {t}^{2}}{\rm{-}}{\rm{i}}\gamma {\left|A\right|}^{2}\right){{A}}=0,$$

Where z is location in the waveguide, *α*_*i*_ is the propagation loss, *ϕL* is the phase detuning per roundtrip, *β*_2_ is the group velocity dispersion coefficient, t is the reference time of the pulse travelling in the cavity and ${\gamma }$ is the nonlinear Kerr parameter. Shifted resonances can be simulated by applying an additional phase shift per roundtrip to the corresponding mode frequencies^26^. To capture the shift applied to the pump resonance, we modify the detuning parameter according to *ϕ* = *ϕ*_0_ + *σδ*_*μ*0_, where *φ*_0_ is the detuning of the laser from the unshifted pump resonance (comb detuning), *σ* is the shift factor applied to the pump, *δ*_*μ*0_ is the Kronecker delta function, and $\mu$ is the frequency mode with *μ* = 0 corresponding to the pump mode.

The normalization for Fig. 2 is performed in the same way as in previous works^36,44^. Concretely, the normalized comb detuning is *Δ*_c_ =*ϕ*_0_*L*/*α*, the normalized pump detuning is *Δ*_p_ = (*ϕ*_0_ + *σ*)*L*/*α* and the normalized power is *X* *=* *P*_in_*Lγ θ/α*^3^, where *α* *=* (*α*_*i*_ *L* + *θ*)/2. The intracavity field and its temporal distribution are also normalized according to $F=A\sqrt{\frac{\gamma L}{\alpha }}$ and ${t}^{{\prime} }=t\sqrt{\frac{2\alpha }{{|\beta }_{2}{|L}}}$, respectively. Note that by changing ${P}_{\rm{in}}$ one can scale the comb to different power levels without change in shape as long as other parameters are adjusted such that *Δ*_0_, *Δp*, *X* and *t*′ remain constant. The converted power in Fig. 2 will remain unchanged with such scaling, unless intrinsic losses are introduced. More details on the existence map simulation are provided in the next section.

The numerical model used for Fig. 3 was based on the coupled-cavity model^36,37^, which captures the nonlinear propagation in both cavities, including pulse walk-off between cavities. To mitigate the mismatch in size between cavities, the auxiliary cavity was scaled to have the same length as the main cavity (2π × 227.8 μm), resulting in a ten times smaller FSR (for example, 97 GHz instead of 970 GHz). However, by only allowing every tenth comb line of the auxiliary cavity to acquire power, we effectively have a spectral distribution of 970 GHz. The coupling between main cavity and auxiliary cavity was only allowed for these selected modes of the auxiliary cavity, and they would interact with the mode main cavity that was closest in frequency.

For Fig. 3, the simulated main cavity had *β*_2_ = −81 ps^2^ km^–1^, an FSR of 99.8 GHz and an intrinsic Q-factor of 10 million. The numerical model has the auxiliary cavity scaled tenfold in the manner described above to represent the 967 GHz cavity. The intrinsic quality factor of the auxiliary cavity is set to 1 million and a GVD of *β*_2_ = −20 ps^2^ km^–1^. The power coupling rate between main cavity and bus was *θ*_t_ = 0.97%, the coupling between cavities was set at *θ*_*c*_ = 0.2141%, and the coupling between auxiliary ring and drop waveguide was *θ*_*d*_ = 0.03%. The nonlinear parameter was based on simulation and set as *γ* = 0.9 (W m)^−1^. The cold-cavity detuning of the laser, and the relative cold-cavity shift between auxiliary resonance and main resonance are provided in Supplementary Fig. 5a. Note that these detuning values do not include the effects of nonlinearity and resonance split.

More details on the relation between the coupled-cavity model and shifted resonance model are provided in the Supplementary Section 6.

### Generating existence maps

To map the existence of DKSs and other comb states in Fig. 2, we conducted a large-scale simulation for different values of power and detuning. The simulations were carried out with *β*_2_ = −20 ps^2^ km^–1^, FSR of 100 GHz, cavity roundtrip length of 1.4313 mm, *θ* = 0.628% and *γ* = 0.9 (W m)^−1^. The input power and detuning are varied to generate a grid of 0.04 resolution of normalized detuning and normalized power. At each grid-point point, the intracavity waveform was propagated up to 80,000 roundtrips so that it would reach a steady-state. After that, 10,000 roundtrips were recorded to characterize the state. Most of the characterization tests that followed were done automatically, with visual verification along the existence map. The converted power of the final intracavity state was calculated to determine if it was a CW state or a comb state. Stability and oscillations were automatically tested by recording the intracavity mean power across these roundtrips. A negligible change in the intracavity mean power would define a stable comb state. An oscillatory mean power was considered to represent a breather, assuming that the cavity contained a single pulse. The number of pulses in the cavity was determined by using the temporal intracavity power, with effects of breathing oscillations removed by averaging from roundtrip to roundtrip, applying peak detection to find the number of pulses in the cavity. A single detected peak was considered to be a single DKS. Multiple detected peaks meant that the comb state was categorized as ‘other’.

The comb states were acquired in three different stages. First the DKS existence was mapped. A single DKS was initiated at some point in the map. The detuning and power were gradually changed to move between grid-points, where the comb state was characterized. The conversion efficiency was defined as the power of output comb lines excluding the pump frequency divided by the input pump power.

A second stage was to observe the presence of modulational instability. Essentially, we did not define modulational instability as a specific comb state, rather presence of parametric gain that enables a CW background to evolve into a comb state. For this, the intracavity field was set to a CW steady state of the cavity (selecting the upper branch in presence of bistability) adding a noise seed of low amplitude. These CW steady-state solutions were found by adapting previously known equations for single cavities^46^, written as $X={Y}^{3}-2{\varDelta }_{\rm{p}}{Y}^{2}+\left({{\varDelta }_{\rm{p}}}^{2}+1\right)Y$, where $X$ is the normalized input power, $Y$ is the normalized intracavity power and ${\varDelta }_{\rm{p}}$ is the normalized pump detuning. In the presence of modulational instability, the CW steady state would evolve into a comb state, typically Turing patterns, chaos or multi-DKS states. The areas which displayed converted power were automatically detected and considered to show modulational instability. In the final stage, starting with intracavity waveforms found at stage 2 as a seed, we tuned to different points of the grid to find potential comb states that might have been missed in the first two stages.

## Online content

Any methods, additional references, Nature Portfolio reporting summaries, source data, extended data, supplementary information, acknowledgements, peer review information; details of author contributions and competing interests; and statements of data and code availability are available at 10.1038/s41566-023-01280-3.

### Supplementary information

Supplementary Information Supplementary Sections 1–9 and Figs. 1–11.

## Data Availability

The raw data of this work can be accessed at 10.5281/zenodo.8117147.

## Code availability

The code is available from the corresponding author on reasonable request.
