# Supplementary material for: Surpassing the nonlinear conversion efficiency of soliton microcombs
Source: Nat Photonics. 2023 Aug 31;17(11):992–9. doi: 10.1038/s41566-023-01280-3 (PMC10618085; doi:10.1038/s41566-023-01280-3)
Supplement: Supplementary file 1 — Supplementary Sections 1–9 and Figs. 1–11. [file 41566_2023_1280_MOESM1_ESM.pdf]

nature photonics

Article https://doi.org/10.1038/s41566-023-01280-3

Surpassing the nonlinear conversion efficiency of soliton microcombs

In the format provided by the authors and unedited

Contents

S1. Analyzing soliton envelope 2
S2. Theoretical comparison of the MI existence area 5
S3. Power scaling of high-conversion efficiency DKS microcombs 6
S4. Consistency of DKS microcomb initiation 8
S5. Simulation of DKSs in coupled cavities 11
S7. Resilience with regards to TOD, coupling and intrinsic Q 13
S8. Other waveforms 16
S9. Backwards initiation of DKS states 18
References 20

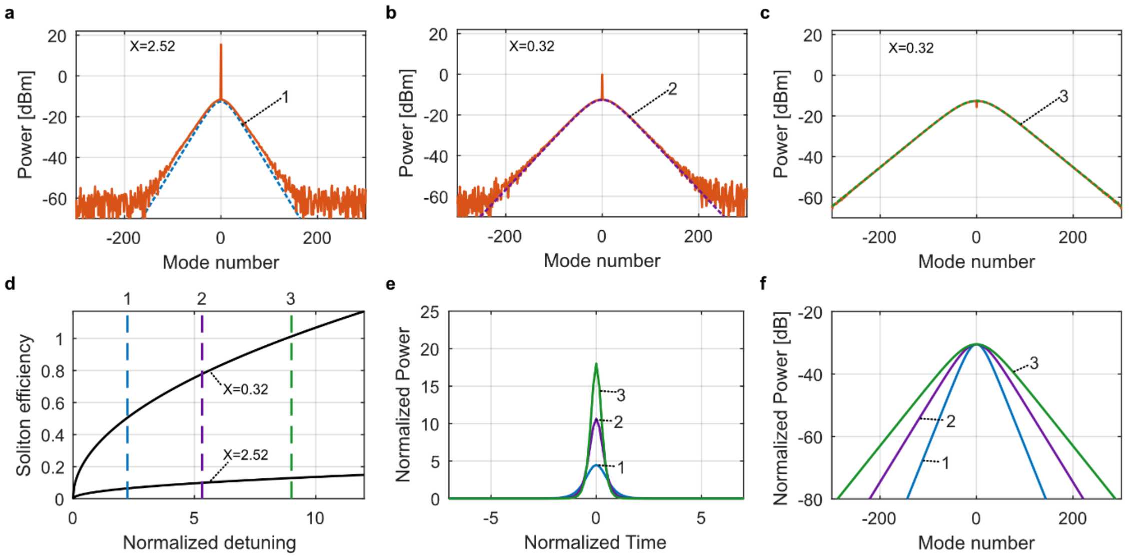

Fig. S1 Analyzing DKS microcomb efficiency using closed-form solutions. a-c The outcoupled spectral distribution of the closed-form solution for detuning 1-3 after transferring to real parameters (dashed), each figure indicating the set power. The red trace in **a** shows the simulation from the anomalous-dispersion cavity in Fig.1a-iii which used the same parameters as the closed form solution. The red trace in **b** shows the simulation from the highest efficiency DKS of the anomalous-dispersion cavity in Fig.2 c, where the pump resonance was shifted by 5 normalized detuning units. The comb detuning in the simulation matches the detuning of the closed-form solution. The red trace in **c** shows a simulation from the shifted anomalous-dispersion cavity in Fig.1b-iii . The comb detuning in the simulation matches the detuning of the closed-form solution. **d**, The change in soliton efficiency according to the closed form solution as a function of detuning for two different levels of normalized Power (X). The efficiency scales with the square root of normalized detuning. The dashed lines mark the detuning of the DKSs in **a-c**. **e** The intracavity field of the soliton for the three detuning levels marked by the dashed lines in **d**. **f** The spectral distribution of the solitons in **e**, showing that the increase in detuning causes the spectrum to get broader.

S1. Analyzing soliton envelope

The high conversion efficiency of DKSs is enabled by high comb detuning and the ability to operate the pump at the center of resonance. Here, we show these dynamics are predicted by the analytical solution for the electric field of the cavity soliton[1,2]. This analytical approximation is written as

$$\mathrm{F}=\sqrt{2\Delta}\,\mathrm{sech}\left(\sqrt{\Delta}t'\right), \qquad (1)$$

where Δ is the normalized detuning, t' is the normalized temporal distribution and F is the normalized intracavity field. These normalized parameters are described further in the method section of the main manuscript. This solution assumes a conservative system, thus it does not depend on power coupled into the cavity or damping factor. It does nonetheless give a good approximation for soliton

waveforms in driven-damped microcavities[3]. As an example, in Fig. S1a, we show a good agreement between the Eq. 1 and the simulation of the outcoupled spectrum from Fig. 1a-iii when using the same parameters.

We find that Eq. 1 can be applied to a cavity with a shifted pump resonance by applying the comb detuning parameter (i.e. by setting $\Delta = \Delta_c$). This is demonstrated in Fig. S1b using the settings of the highest efficiency DKS in Fig. 2c, where the main resonance had been shifted by 5 normalized units. The result shows a remarkable agreement between Eq. 1 and simulation, showing that the DKS shape is determined by the comb detuning. We evaluate the results again in Fig. S1c using the same setting as in Fig. 1b-iii, operating the comb with comb detuning $\Delta_c = 9$, pump detuning $\Delta_p = 0.25$ and normalized power $X = 0.32$, resulting in >99% conversion efficiency.

Having established the closed-form expression in Eq. 1 as a useful qualitative approximation for DKSs in anomalous-dispersion cavities with shifted pump resonances, we investigate the scaling of conversion efficiency. Integrating Eq. 1 we find the soliton energy as $E_{cs}=4\sqrt{\Delta}$. Dividing the soliton energy with energy input per round trip, we find the soliton efficiency as

$$\eta_{cs}=\frac{\theta^2 E_{cs}}{\alpha^2 X\,T}=\frac{\theta^2 4\sqrt{\Delta}}{\alpha^2 X\,T}, \qquad (2)$$

which becomes $\eta_{cs}=\frac{16\sqrt{\Delta}}{X\,T}$ in absence of intrinsic losses, where X is the normalized power, T is the normalized roundtrip time, θ is the coupling rate between bus and ring and α is the cavity loss per roundtrip. Note that η_{cs} gives a good approximation of the conversion efficiency for our purposes, but it has a slightly different definition, where 100% soliton efficiency means that the pump has been transferred perfectly into a hyperbolic secant shape. Equation 2 should be treated as an approximation as it does not take into account pump depletion, thus higher than 100% conversion efficiency can be predicted, which is of course not realistic.

We plot the soliton efficiency with regards to detuning in Fig S1.d, with the waveforms of the closed form solutions for selected detuning values (labelled 1-3) plotted in Fig S1.e-f. The normalized roundtrip time of the cavity was the same as in Fig. 2b-c. The figures emphasize the dramatic improvement that can be made in conversion efficiency by decreasing the normalized power from 2.52 to 0.32 and by increasing the normalized detuning. Unfortunately, as was illustrated in Fig. 2b, such improvements are not available in isolated anomalous-dispersion cavities (i.e. without shifted pump resonance). The reason for this limitation is that increased detuning leads to the pump being

operated far away from resonance center such that it is inefficiently coupled into the cavity. This ultimately sets a maximum detuning (and minimum power) limit at $\Delta = \pi^2 X/8$ as has been discussed in previous work[1,2]. The presence of modulational instability (MI) also eliminates the possibility to operate the DKS with the pump near the center of resonance. Thus, conversion efficiency is fundamentally limited in unperturbed anomalous-dispersion cavities, as illustrated by our simulations in Fig. 2b which found the peak conversion efficiency at $X = 2.52$ and $\Delta = 2.24$, with the simulated comb spectrum displayed in Fig S1a.

The purpose of shifting the pump resonance is to separate the detuning of the pump from the detuning of the comb. This way, we overcome the upper limit set on the comb detuning, allowing us to scale up the converted power by increasing the number of lines. A second consequence is that the DKS can be operated with the pump at the center of resonance at a significantly lower power, possibly due to reduced modulational instability (see section S2). The combination of these two factors allows the DKS to be operated with extremely high efficiency, as is predicted by Fig. S1d. This allows us to reach the low normalized power of $X = 0.32$ and a high detuning as is shown in Fig. S1b-c.

The soliton efficiency in equation 2 is also dependent on another parameter, the normalized roundtrip time defined as $T = \frac{1}{FSR}\sqrt{\frac{2\alpha}{|\beta_2|L}}$. Thus, it can be tuned in practice by changing the GVD (β_2). Tuning this parameter not only impacts the conversion efficiency, but also the number of lines. By doing Fourier analysis on Eq. 1 we find that the 3 dB drop off point of the soliton is found at $f_{3db} = \frac{0.8814\sqrt{\Delta}}{\pi^2}$, where f is the normalized frequency. The number of lines is found as $N = \frac{2f_{3db}}{f_{FSR}} + 1 = 2\frac{0.8814\sqrt{\Delta}}{\pi^2}T + 1$, where $f_{FSR} = 1/T$ is the normalized FSR. The added one line is to include the pump line which is in the middle of the spectrum.

It thus becomes clear that in an unperturbed anomalous-dispersion cavity, where the detuning is limited to $\Delta = \pi^2 X/8$, there will be a trade-off between the number of lines and conversion efficiency. The soliton conversion efficiency[4] will scale as $\eta_{cs} \propto 1/N$ (since $N \propto T$ and $\eta_{cs} \propto 1/T$). However, this limitation is lifted when the pump resonance is allowed to be shifted, as we can select Δ more freely. With careful engineering, we can in principle find the correct combination of Δ, X and T to generate high efficiency DKS for a pre-determined number lines as we find in section S3. It is then up to the

designer to translate the normalized parameters into realistic designs depending on requirements and fabrication constraints.

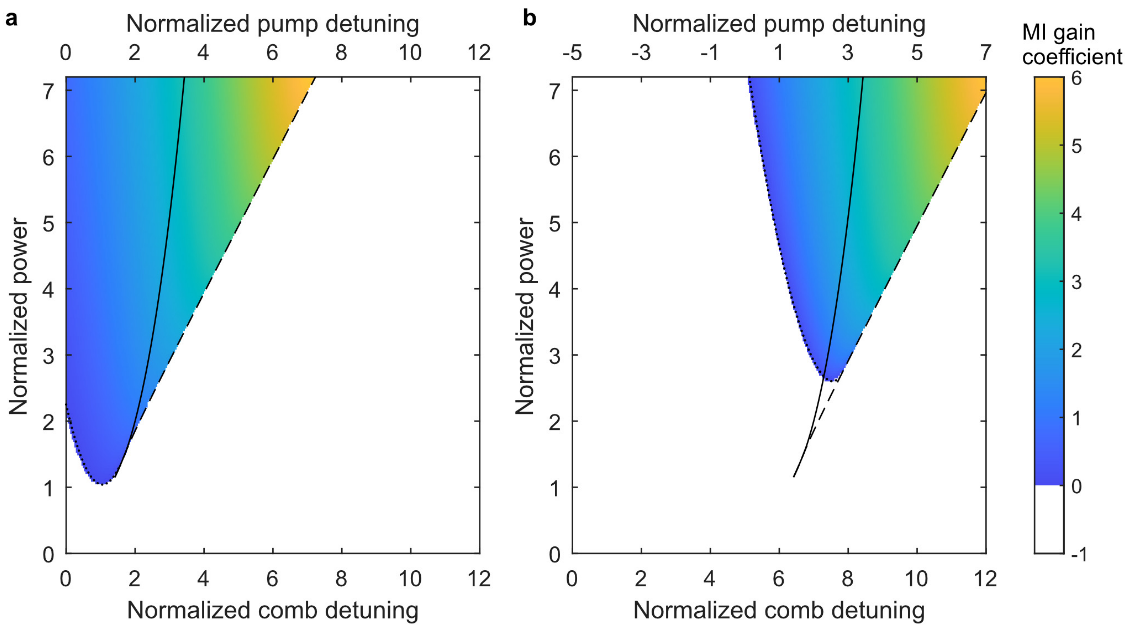

Fig. S2 MI gain coefficient estimated from theory. **a**, The color grading shows the maximum MI gain coefficient acquired from analytical equation for a cavity with an unshifted resonance. The dashed line and solid line confine the area of bistability. The dotted line and the dashed line confine the area where MI was found in simulations. **b**, Same as **a**, but with the pump resonance shifted by 5 normalized unit, same as in Fig.2c. The gain existence acquired from the analytical equation matches well the MI area found in our simulations.

S2. Theoretical comparison of the MI existence area

A highly interesting result from Fig. 2c is that the modulational instability is limited to higher power levels. Here we analytically investigate these results using the gain coefficient derived for small electric-field perturbations in a CW driven cavity[5]. The gain coefficient is written as

$$\lambda = -1 \pm [4(\Delta - \xi\omega^2)Y - (\Delta - \xi\omega^2)^2 - 3Y^2]^{1/2}, \qquad (2)$$

where ξ is the sign of the GVD (set to -1 for anomalous dispersion), ω is the normalized radial frequency and Y is the normalized intracavity power. In the presence of a shifted pump resonance the detuning is set to equal the normalized comb detuning, $\Delta = \Delta_c$. We set the intracavity power by solving the CW steady-state cubic equation $X = Y^3 - 2\Delta_p Y^2 + (\Delta_p{}^2 + 1)Y$, where Δ_p is the normalized

pump detuning. We use these equations to numerically find the maximum gain coefficient with regards to ω for the existence maps in Fig. 2b-c. Fig. S2a shows the results for the unshifted cavity from Fig. 2b, while Fig. S2b shows the shifted cavity from Fig. 2c, both showing excellent agreement with the simulations. These results show that the MI dynamics can be predicted using the pump detuning to define the CW intracavity build-up and the comb detuning to predict the interaction between comb lines. The high comb detuning results in reduced phase matching which limits the MI to higher normalized power levels. This absence of MI at lower power might be the reason why DKSs are allowed to exist near the center of pump resonance in Fig. 2c.

S3. Power scaling of high-conversion efficiency DKS microcombs

The existence map in Fig. 2c shows that the normalized power needs to be reduced significantly to reach high-efficiency DKS-states. However, this does not restrict these waveforms to low input power, as the normalized power involves several parameters. In this section, we demonstrate via simulations how the conversion efficiency of the photonic molecule DKS microcombs can be maintained above 99% while increasing the pump power over 10 dB by simply adapting the comb detuning, pump detuning, GVD coefficient and coupling rate to the bus waveguide, where the latter two parameters can be engineered with lithographic control in silicon nitride. We also provide an experimental demonstration based on a different photonic molecule design that demonstrates that high conversion efficiency can be obtained with largely dissimilar power levels.

When increasing the input power, other parameters can be adjusted to maintain the same normalized values. This is demonstrated in Fig. S3a-b, which shows an example of how the comb can be operated at higher CW input power (P_{in}), while maintaining the same relative comb bandwidth and conversion efficiency, and drastically changing the pump power by more than an order of magnitude. These combs were generated with the numerical model used in Fig.2, but with a higher shift to the pump resonance, allowing near 99% conversion efficiency. As the input power is increased (Fig. S3a), the coupling, pump detuning, comb detuning and GVD coefficient are all increased in the same proportion to maintain the same normalized pump power.

Fig. S3c-d reveal that high efficiency solitons can be generated at different comb bandwidths. Fig. S3c shows the scaling of parameters as the input power is increased, with the GVD coefficient kept

constant. The corresponding comb states demonstrate an increase in power and number of lines, maintaining the 99% conversion efficiency. Note that the parameter scaling does not keep the normalized parameters (X, Δ_c, Δ_p and t') fixed, instead a new combination is found in each case to allow the increased number of lines. This change is expected according to our analysis of number of lines in section S1. We also note that realizing higher comb detuning will require a larger resonance shift. When realizing the design in coupled cavities, this means that the coupling between cavities need to be strong enough to realize the desired shift.

The scaling in Fig. S3a and Fig. S3c shows that increasing the coupling factor can be modified to scale high efficiency comb states to a higher or lower power level. To demonstrate this, we fabricated a second device with lower coupling rate by placing the main cavity further from the bus waveguide, resulting in extrinsic Q-factor of 2.5 million. Using the same initiation methods as in Fig. 3, we generate a DKS state measured with 51% conversion efficiency (see Fig. S3e). The estimated on-chip CW power was 5 mW, resulting in roughly 2dB decrease in total comb power compared to Fig. 3d.

The device in Fig. S3e had a facet coupling loss of ~3dB per facet. It had a main cavity of radius 227.82 μm and the radius of the auxiliary cavity was 23.32 μm. All waveguides had the same dimensions of 1800 nm width and 740 nm height. The gap between rings was 500nm, the gap between main cavity and bus was 400nm. The main cavity was measured (referenced to 1566 nm) with GVD of $\beta_2 = -89\,ps^2/km$, FSR of 99.72 GHz, intrinsic Q factor with an average near 8 million, and a coupling rate to bus waveguide corresponding to extrinsic Q of 2-3 million near 1566nm. The maximal resonance shift induced by the auxiliary cavity was measured as 770 MHz. The auxiliary cavity resonance at 1566.3 nm which interacted with the main cavity had 1.2 million intrinsic Q and coupling rate to a bus waveguide corresponding to 50 million of extrinsic Q. The auxiliary FSR was 969.5 GHz.

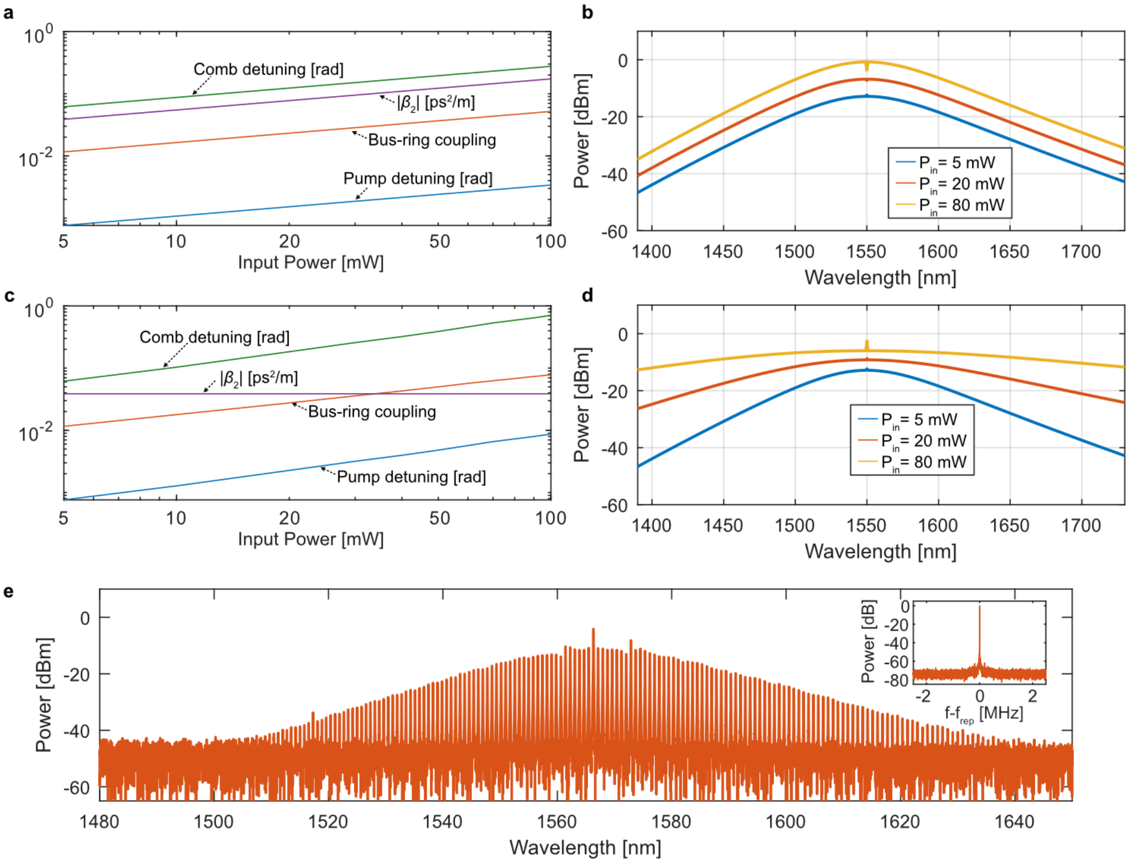

Fig. S3 Universal scaling of high efficiency solitons. **a**, For each input power level, the displayed parameters are scaled in order to maintain >99% conversion efficiency without changing the comb envelope, with three corresponding comb distributions shown in **b**. **c**, An example of parameter tuning to change comb bandwidth while maintaining 99% conversion efficiency, with three corresponding comb distributions shown in **d**. **e**, A comb spectrum acquired from a device which has slightly lower coupling factor between bus and ring compared to the device used in Fig. 3, resulting in 51% conversion efficiency. The inset shows the downconverted repetition-rate beat note, measured with 100Hz resolution bandwidth, indicating a coherent comb state.

S4. Consistency of DKS microcomb initiation

The initiation of DKS states in the coupled cavity system was observed to be highly reproducible, with the majority of initiation attempts resulting in a single-DKS state. To demonstrate this, we used an arbitrary waveform generator (AWG) to control the laser frequency, auxiliary heater and laser power for the DKS initiation steps, as described in Fig.3 of the main manuscript. This allowed us to make a pre-programmed control voltage trace for the initiation, to demonstrate the repeatability of the initiation

process, with adjustable initiation speed. The setup used for this test is shown in Fig. S4a. The setup allows the pre-programmed voltage traces to be measured synchronously with the transmitted and converted power from the microcavities. Fig. S4b shows a measurement of controlled voltages for the initiation steps. Fig. S4c shows the resulting evolution of the converted power and transmitted power after running the initiation program 5 consecutive times, with each initiation process taking roughly 100 seconds. The resulting traces have remarkable similarity, each resulting in a high-efficiency DKS microcomb state. Fig. S4d shows a similar test, with initiation process running 25 consecutive times with 20 times faster speed. The corresponding converted and transmitted power show high repeatability. Overall, these experiments indicate that the high-efficiency DKS in our coupled cavity system can be generated in a reliable manner, without the need to precisely control the tuning speed of the instruments.

The initiation strategy was found from insights from simulations in Fig. 2 of the main manuscript and simulations in the next section. The exact tuning of laser frequency and auxiliary resonance location was typically done with a 10 MHz resolution. A study into the robustness and long-term stability of the DKS was not carried out, as the lensed fiber alignment setup used to couple the microchip was typically not stable for more than 30 minutes at a time.

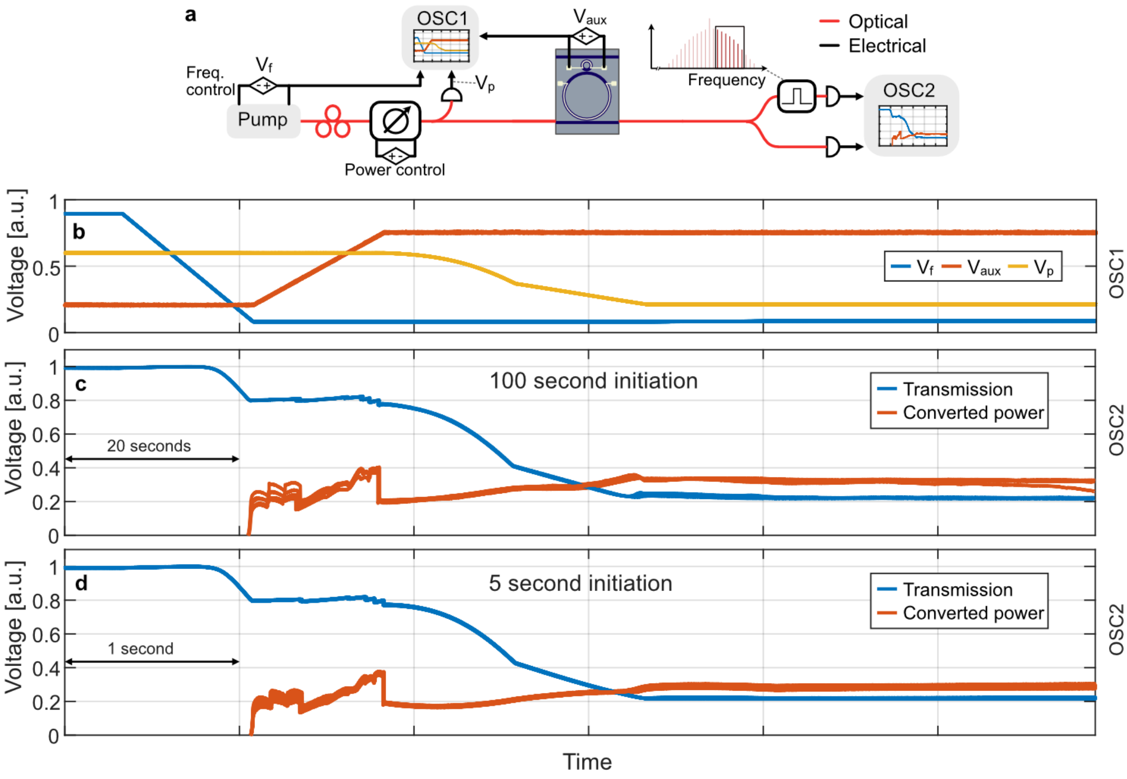

Fig. S4 Automated control and consistent initiation of a high-efficiency single-DKS microcomb a, The setup used to initiate a soliton using a pre-programmed pathway in terms of laser frequency, auxiliary heater voltage and laser power. The corresponding control voltages (V_f, V_{aux} and V_p) are measured in oscilloscope 1 (OSC1). The signals were provided using an arbitrary waveform generator (AWG). **b** An oscilloscope measurement of the control voltages. Period of the sequence could be stretched by re-configuring the AWG. **c** The initiation evolution towards of a single-DKS state in terms of transmitted power and converted power using a 100s long sequence. The initiation was run 5 consecutive times, each time reaching a single-DKS state. **d** The result of applying the same initiation sequence, but 20 times faster. The figure shows 25 consecutive initiation attempts, each of them resulting in a single-DKS state.

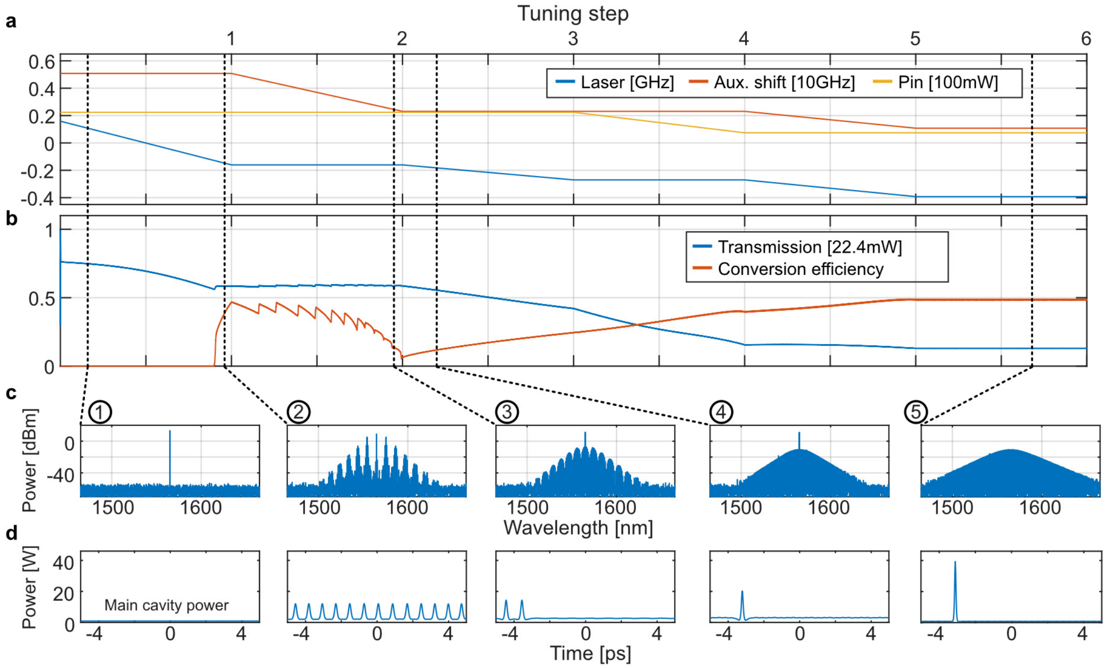

Fig. S5 Scaling of high efficiency solitons. a, The tuning steps in terms of the control parameters; laser frequency, auxiliary resonance location, and input power that result in high efficiency soliton. The laser frequency and auxiliary location are given as frequency offset from the unshifted main-cavity resonance. Each step consists of 500,000 main cavity roundtrips. **b** The response to the tuning steps in terms of conversion efficiency and transmitted power. **c** The output power spectrum found at selected points (indicated by dashed lines) in **b. d** The temporal power distribution of the main cavity, corresponding to the spectra in **c**.

S5. Simulation of DKSs in coupled cavities

The initiation dynamics of a high-efficiency DKS in our coupled-cavity device can be qualitatively replicated using numerical simulations. The simulated initiation was conducted using a step-by-step tuning of the laser frequency, auxiliary resonance and input laser power similar to the experiment in Fig. 3. Fig. S5a shows how these parameters are tuned, and Fig. S5b shows the response in terms of transmitted power and conversion efficiency. We start with the auxiliary resonance far on the blue side and the laser frequency slightly blue-detuned from the main cavity resonance, with a CW state in the main cavity as indicated by the output spectrum and intracavity power of comb state 1 in Fig. S5c-d. The first step is to tune the laser towards the red side until a Turing-roll is generated, as indicated by the temporal power distribution of state 2 in Fig. S5d. The next step is to tune the auxiliary resonance closer to the main resonance induce a stronger resonance split, effectively shifting the pumped resonance. The evolution of the conversion efficiency during this tuning step shows a series

of step-like transitions. These transitions correspond to the reduction of pulses in the main cavity, eventually leading to a two-soliton state (i.e. comb state 3) and then a single soliton state (comb state 4). The next series of steps involve reducing the pump power, much like in Fig.3, with additional tuning of auxiliary resonance location and laser frequency. The final state is a DKS state with 48% conversion efficiency.

The intracavity field of the final waveform is displayed in Fig. S6. The spectral power in the main cavity has a shape that is close to a hyperbolic secant, with visible deviants in locations where mode-crossings occur with the auxiliary cavity. The CW component has a high-power level in the main cavity which feeds comb lines at the other frequencies through nonlinear processes.

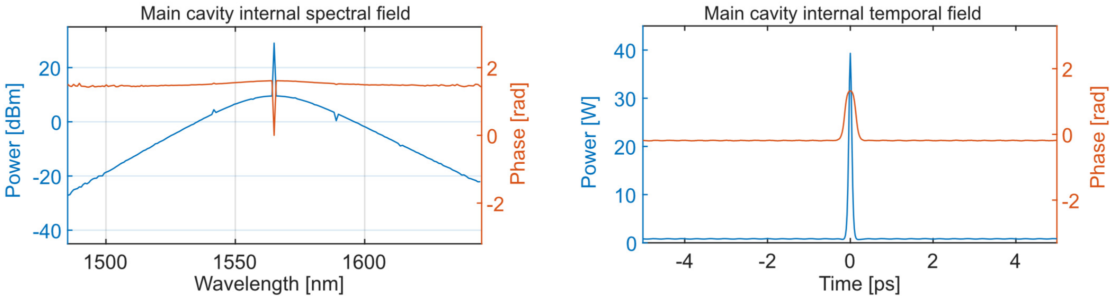

Fig. S6 Detailed intracavity field. The distribution of power and phase of the main internal cavity, both in the spectral domain and in the time domain.

S6. Comparing the coupled-cavity model with the shifted-resonance model

The physics of two linearly-coupled cavities (or linearly-coupled transverse modes) with nonlinear dynamics can be difficult, especially when we consider the fact that the number of parameters has been approximately doubled compared to a system with a single cavity. In this work, we find that nonlinear dynamics only occur in the main cavity, with an optical field of low power in the auxiliary cavity inducing linear perturbations to select longitudinal modes of the main cavities. These perturbations result in the appearance of split eigenmodes which are shifted compared to the longitudinal modes of the isolated cavities[6]. In previous studies, the effects of such perturbations on microcomb generation has been accurately captured by approximating the impact of the auxiliary cavity as fixed frequency shift applied to selected longitudinal modes of the main cavity[7–10]. For a perturbation from a lossless auxiliary cavity that only occurs at the CW pumped resonance, this leads to the shifted resonance model used in Fig. 2. The impact of auxiliary resonance losses can be included by modifying the quality factor of the pumped resonance[11].

To demonstrate the link between the coupled-cavity model and the shifted resonance model, we use the comb state found in the coupled cavity simulation of Fig. S6. The intracavity fields were observed to be steady state, with auxiliary cavity causing a fixed phase shift and fixed amount of losses to pump frequency component. We replicate the same results using a single cavity simulation with the pumped resonance red shifted by 355.7 MHz, and the intrinsic quality factor of the pumped resonance modified to be 2.92 million. The mode-crossings at other frequencies were ignored, as they do not have a critical impact on the overall comb state. The results show that the shifted resonance model closely replicates the coupled-cavity model when generating the high efficiency soliton. The strong agreement between the models validates the replacement of the auxiliary cavity with a shifted resonance.

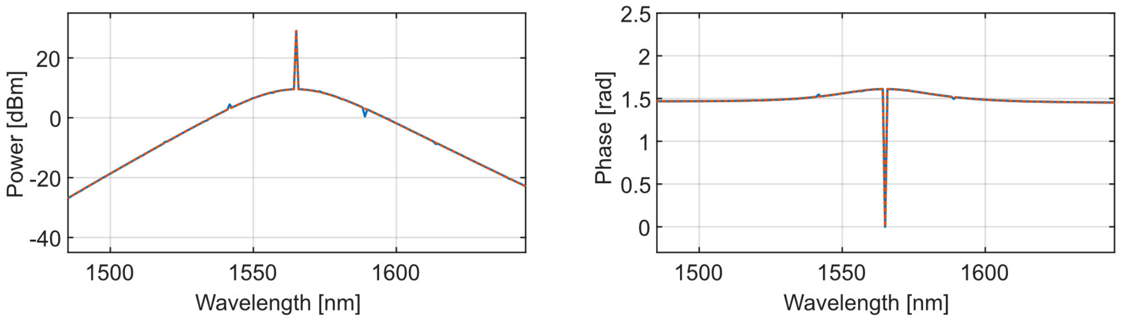

Fig. S7 Comparing coupled-cavity simulations with shifted resonance simulations. The blue trace shows a simulation of the main cavity spectral field using the coupled-cavity model. The red trace shows a simulation where the auxiliary cavity was replaced with a fixed shift and extra loss to the pumped resonance. Apart from the mode-crossings at other frequencies, we observe an almost exact match between the two models, both in terms of power and phase.

S7. Resilience with regards to TOD, coupling and intrinsic Q

In this section we do a brief numerical investigation into the impact of the third order dispersion (TOD), coupling between cavities and the intrinsic Q factors.

We first test the resilience with regards to TOD, which can affect the existence of solitons[7,12]. We introduce TOD with $\beta_3 = \pm 5\ ps^3/km$, using the soliton simulation from Fig. 3d with other parameters unchanged, except the input power had to be increased to 9 dBm to maintain the comb. The resulting spectrum is shown in Fig. S8a-b, displaying a dispersive wave at 44% conversion efficiency. This test suggests that high efficiency solitons can be attained in the presence of dispersive waves, which could be useful when operating DKS states with an octave spanning spectrum.

The second test involves investigating the impact of the quality factors of the main and auxiliary cavity. We again use the numerical simulation from Fig. 3d. In Fig. S8c, we removed the intrinsic losses of the main cavity. This allowed the comb to be operated at a lower pump power of 7.4 dBm, resulting in an increased conversion efficiency of 65%. In Fig. S8d, we also remove the losses of the auxiliary cavity, such that there are no intrinsic losses in the system. This allows us to operate at an even lower pump power of 6dBm, with the comb state reaching almost unity conversion efficiency. For the system with no intrinsic loss, the laser offset from cold cavity resonance was set to 495.6 MHz on the red side, and the location of uncoupled auxiliary resonance was located 627.9 MHz towards the blue side of the main resonance. This suggests that the near unity conversion efficiency demonstrated in Fig.1 of the main manuscript can indeed be attained in coupled cavities, assuming the intrinsic losses can be mitigated.

Finally, in Fig. S8e-f we drastically change coupling between main and auxiliary cavity (θ_c), by both increasing and decreasing the coupling by a factor of 4. Other parameters of the simulation used the same parameters as in Fig. 3d, except the input power, laser frequency and auxiliary resonance location (i.e. the parameters we can control in an experimental setting) had to be shifted to account for the change in coupling. Fig. S8e shows the result of increasing the coupling by a factor 4. The input power was kept at 8.7 dBm, with the laser detuned 460.6 MHz on the red-side from the main cold-cavity resonance location and the uncoupled auxiliary resonance shifted 4.156 GHz towards the blue side of the main resonance. The result was an improved conversion efficiency of 63%. This improvement occurs since with a stronger coupling strength less power needs to be built up in the auxiliary cavity achieve the desired resonance shift. Since the auxiliary cavity is lossy (Q=1 million), this means that the overall losses are decreased.

Fig. S8f shows the result of decreasing the coupling by a factor 4. The input power was set to 10.8 dBm, with the laser detuned 285.9 MHz on the red-side from the main cold-cavity resonance location and the uncoupled auxiliary resonance shifted 329.7 MHz towards the blue side of the main resonance. The result was a conversion efficiency of 26%. This reduction in conversion efficiency occurs partially because of higher effective losses to the pump, as more of it is coupled to the auxiliary cavity. The other reason is that the lower coupling factor does not achieve sufficient resonance shift, such that we cannot reach a point where we have fully converted the remaining pump power to the other comb lines.

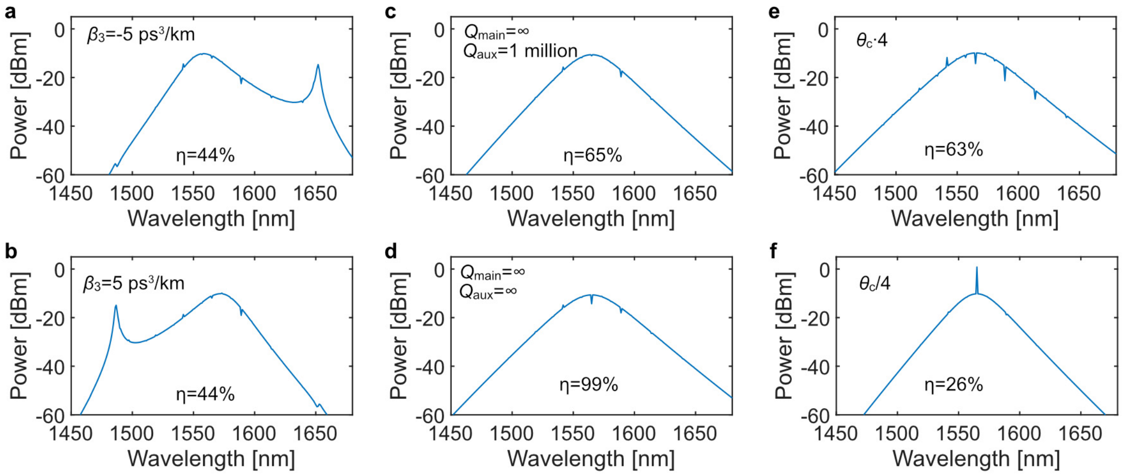

Fig. S8 Resilience to parameter variations. **a-b** The outcoupled comb power of a soliton with a strong third order dispersion. **c** An outcoupled spectrum achieved when turning off the intracavity losses of the main cavity. **d** An outcoupled spectrum with no intrinsic losses in the coupled cavities. **e** An outcoupled spectrum achieved with 4 times higher power coupling between the two cavities. **f** An outcoupled spectrum achieved with 4 times lower coupling between the two cavities. The conversion efficiency is given by η in each figure.

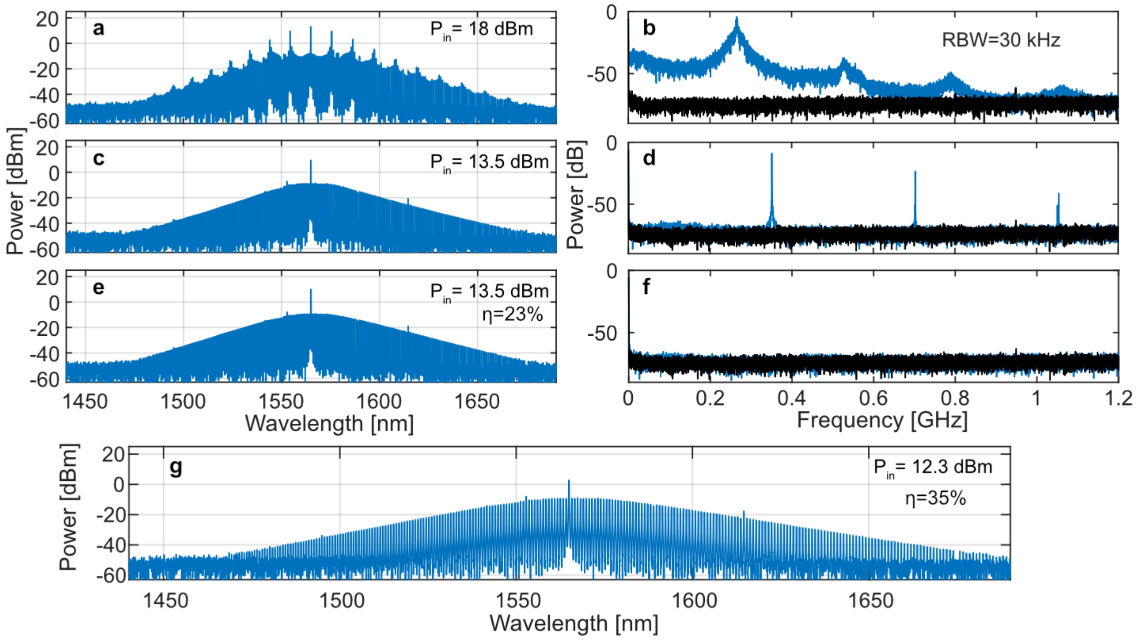

Fig. S9 Various comb states found in the photonic molecule. a An OSA spectrum measurement of a microcomb state initiated at high power. **b** The blue trace shows the corresponding transmitted power measured in a electrical spectrum analyser (ESA) through a photodiode. The black trace shows a reference trace recorded with no comb being generated. The resolution bandwidth (RBW) was set to 30kHz. The increased power over a wide spectrum suggests that this might be a chaotic state. **c** An OSA spectrum showing a state which has near hyperbolic secant shape. **d** The throughput of the state measured in the ESA shows that the output oscillates at specific frequencies, which suggests that this is a breather state. **e-f** A single DKS state with no excess noise in the ESA measurement, indicating a stable soliton state with 23% conversion efficiency. **g** A single DKS state initiated at 12.3 dBm (i.e. without reducing the input power), with 35% conversion efficiency.

S8. Other waveforms

The existence map of Fig. 2c in the main manuscript shows the presence of microcomb waveforms with various characteristics. This includes blue and red detuned DKS states, breather solitons, chaotic states. Here, we demonstrate some of the states that were discovered outside of the initiation process described in the main manuscript. In Fig. S9a, we show a microcomb state recorded in an OSA at high pump power, with Fig. S9b showing the transmitted power measured in an electrical spectrum analyser through a photodiode. The raised radio frequency background indicates an incoherent state, potentially occurring from a chaotic state. We did not observe such chaotic states at lower input power levels. In Fig. S9c, we show a single-DKS state, which was recorded at 13.5 dBm of input power. The initiation of this soliton was the same as in Fig. 3b, except instead of the power reduction we tuned the laser further into resonance. The ESA spectrum of the transmitted power (see Fig.S9d), shows

clear signs of oscillations, indicating that the waveform is possibly a breather soliton. By further modifying the auxiliary heater voltage and the laser frequency, we find that we can get rid of these oscillations, resulting in a stable DKS state with 23% conversion efficiency (see Fig. S9e-f).

We also found that we could initiate a DKS state starting with a power level of 12.3 dBm, using feedback locking to stabilize the transition between soliton states. Fig.S9g shows a single DKS state operated at 12.3 dBm power with 35% conversion efficiency. This was the highest efficiency attainable in our devices when keeping a constant power level through the initiation process. The stable solitons of Fig. S9e-g show how the conversion efficiency is reduced as we move to a higher pump power, a feature that was predicted by the existence map in Fig. 2c.

The existence map in Fig. 2c shows that the single-DKS states exist on the red side, and on the blue side of the shifted pump resonance in a continuous stretch. Here, we verify the existence of both states experimentally using the DKS state of Fig. 3d. To test this, we use the counterpropagating probe setup from Fig. 3a. We transit the pump from the blue-detuned side to the red-detuned side of the resonance by simply shifting the pump frequency. The transmission as a function of frequency is displayed in Fig. S10a and the corresponding comb state shown in Fig. S10b. The probe trace shows the two hybridized resonances with the pumped resonance located on the red side. The pumped resonance exhibits a clear beat note between the probe laser and pump laser appearing on the blue side of resonance, indicating that the comb is indeed operated with a blue-detuned laser.

When the pump frequency is tuned towards the red side of the resonance, it eventually results in the beat note flipping to the red side of resonance with a slight change in the spectral distribution of the comb (see Fig.S10c-d). This suggests that we are operating at a point similar to the bottom of the DKS existence in Fig. 2c, where blue-detuned and red-detuned DKSs exist in a continuous stretch. From Fig. S10b and Fig. S10d, we can see that the power of the CW power changes significantly when tuning the laser frequency. This is due to the fact that the cavity is close to being effectively critically coupled from the perspective of the CW frequency. The contributors to the effective intrinsic losses of the CW frequency are the conversion of power to other frequencies, main cavity intrinsic losses and intrinsic auxiliary cavity losses. Tuning the laser frequency will change the conversion efficiency, making the cavity more (or less) critically coupled. This leads to sharper variations in the pump component.

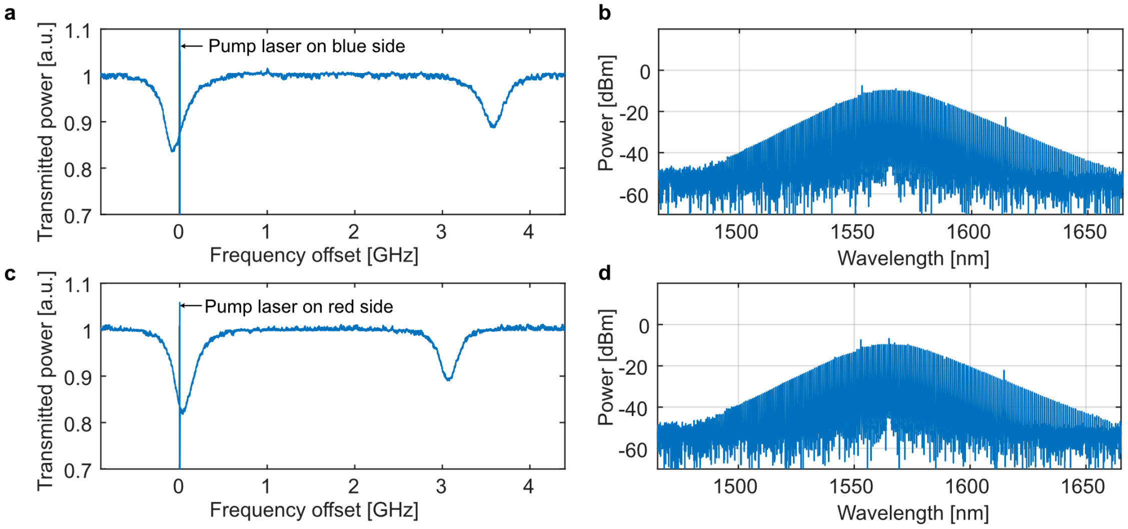

Fig. S10 Microcombs operated on either side of shifted resonance. a The response of the counter-propagating probe-laser, showing that the beating with the pump laser occurs on the blue side of cavity resonance. **b** The power spectral distribution of the corresponding comb measured in an optical spectrum analyzer (OSA). **c** The response of the counter-propagating probe-laser after shifting the pump frequency to the red side of the resonance. **d** shows the corresponding comb state as measured in the OSA.

S9. Backwards initiation of DKS states

With high enough input power, Fig. 2c anticipates that blue-detuned DKSs exist on the blue side of MI, indicating that the DKSs can be initiated by tuning the CW laser into resonance from the red side towards the blue, a phenomenon not found in unperturbed anomalous-dispersion cavities. We execute a series of experiments and simulations to verify these dynamics using the device presented in Fig. S3e which has characteristics similar to the device in Fig.3. The FSR is 99.72 GHz and group velocity dispersion (GVD) coefficient $\beta_2 = -89\ ps^2/km$. The FSR of the auxiliary cavity was 969.5 GHz. The heaters of the cavities were set such that a resonance split appears near 1566.3nm. Here, we conduct a numerical simulation based on measured device parameters. Figure S11a-b show the results both for simulated and measured oscilloscope scans of the transmission and converted comb powers as the laser is swept up in frequency, starting on the red side of the resonance. The auxiliary heater is kept at a fixed level and the on-chip input power is estimated to be 10.6 dBm. The transmission trace displays the main resonance on the red side which is influenced by the proximity of

the auxiliary resonance. The trace of converted comb power indicates a comb is generated when pumping the shifted main resonance. A close inspection of Fig. S11c-d shows that the converted trace exhibits soliton steps, with good qualitative agreement between simulation and measurement. Thermal effects were not included in the simulation, which will cause some quantitative mismatch compared to the measurement. Stopping the laser on the soliton step, we encounter a single-DKS state (see Fig. S11e-f). This state was found to be located on the blue side of resonance, where a reduction in pump power can be used to achieve a DKS with 51% conversion efficiency (See supplementary S3). The fact that we end up with a blue-detuned soliton is a unique feature of this arrangement, not to be confused with other methods that enable red-side operation with a blue-detuned auxiliary laser[13], or the photorefractive effect[14]. This approach could also be accomplished with the device used in Fig. 3. We note that this backwards initiation was in general more sensitive compared to the initiation method described in Fig. 3, as the auxiliary cavity heater needed to be set more accurately.

The main cavity of the simulation had an intrinsic quality factor of 10 million, extrinsic quality factor of 2.5 million, GVD of $\beta_2 = -89\, ps^2/km$ and FSR of 99.72 GHz. The second cavity had quality factor of 1.2 million and dispersion of $\beta_2 = -89\, ps^2/km$. The numerical model has the auxiliary cavity scaled tenfold in the manner described above to represent the 970 GHz cavity. We observed that the dispersion of the second cavity had virtually no impact on our simulations. The nonlinear parameter of both cavities was set to $\gamma = 0.9\ (W\ m)^{-1}$. The ratio of power coupled between the two cavities each roundtrip was 0.24%. The laser frequency describes detuning from main resonance with 873 MHz added. The auxiliary resonance was detuned 2.856 GHz towards the blue side of the main resonance.

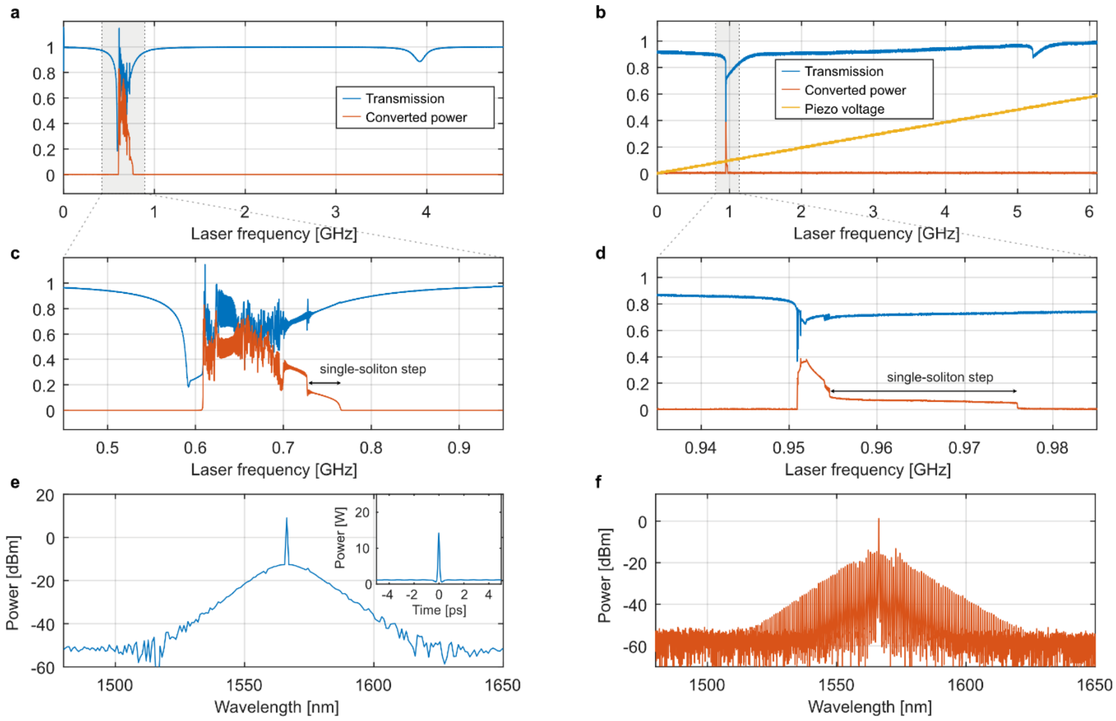

Fig. S11 Backwards initiation of solitons. a A simulated scan of laser frequency, tuning across the coupled resonances from the red side, i.e. starting from the left and moving towards the right. The scan reveals the main resonance (left side) which is slightly shifted due to the proximity of an auxiliary resonance (right side). The converted power rises in the main resonance where a comb is generated. **b** shows a measurement using the same configuration. The laser frequency is swept up by increasing the piezo voltage of the laser. **c-d** A close-up of the simulated and measured laser scan, revealing a soliton step. **e-f** The simulated and measured soliton acquired when stopping the laser on the soliton step. The inset in **e** shows the main-cavity temporal field.

References

1. Coen, S. & Erkintalo, M. Universal scaling laws of Kerr frequency combs. *Opt. Lett.* **38**, 1790–1792 (2013).
2. Barashenkov, I. V & Smirnov, Y. S. Existence and stability chart for the ac-driven, damped nonlinear Schrödinger solitons. *Phys. Rev. E* **54**, 5707–5725 (1996).
3. Herr, T. *et al.* Temporal solitons in optical microresonators. *Nat. Photonics* **8**, 145–152 (2014).
4. Bao, C. *et al.* Nonlinear conversion efficiency in Kerr frequency comb generation. *Opt. Lett.* **39**, 6126–6129 (2014).
5. Haelterman, M., Trillo, S. & Wabnitz, S. Dissipative modulation instability in a nonlinear

dispersive ring cavity. *Opt. Commun.* **91**, 401–407 (1992).

6. Haus, H. A. & Huang, W. Coupled-mode theory. *Proc. IEEE* **79**, 1505–1518 (1991).
7. Herr, T. *et al.* Mode Spectrum and Temporal Soliton Formation in Optical Microresonators. *Phys. Rev. Lett.* **113**, 123901 (2014).
8. Xue, X. *et al.* Mode-locked dark pulse Kerr combs in normal-dispersion microresonators. *Nat. Photonics* **9**, 594–600 (2015).
9. Jang, J. K. *et al.* Dynamics of mode-coupling-induced microresonator frequency combs in normal dispersion. *Opt. Express* **24**, 28794–28803 (2016).
10. Yu, S.-P. *et al.* Spontaneous pulse formation in edgeless photonic crystal resonators. *Nat. Photonics* **15**, 461–467 (2021).
11. Xue, X. *et al.* Normal-dispersion microcombs enabled by controllable mode interactions. *Laser Photon. Rev.* **9**, L23–L28 (2015).
12. Yao, B. *et al.* Gate-tunable frequency combs in graphene–nitride microresonators. *Nature* **558**, 410–414 (2018).
13. Zhou, H. *et al.* Soliton bursts and deterministic dissipative Kerr soliton generation in auxiliary-assisted microcavities. *Light Sci. Appl.* **8**, 50 (2019).
14. He, Y. *et al.* Self-starting bi-chromatic LiNbO 3 soliton microcomb. *Optica* **6**, 1138 (2019).
